# Isoliquiritigenin decreases the incidence of colitis-associated colorectal cancer by modulating the intestinal microbiota

**DOI:** 10.18632/oncotarget.13347

**Published:** 2016-11-15

**Authors:** Minna Wu, Yaqi Wu, Baoguo Deng, Jinsong Li, Haiying Cao, Yan Qu, Xinlai Qian, Genshen Zhong

**Affiliations:** ^1^ Laboratory of Cancer Biotherapy, Institute of Neurology, the First Affiliated Hospital of Xinxiang Medical University, Xinxiang, Henan, China; ^2^ College of Basic Medicine, Xinxiang Medical University, Xinxiang, Henan, China; ^3^ Henan Collaborative Innovation Center of Molecular Diagnosis and Laboratory Medicine, Xinxiang Medical University, Xinxiang, Henan, China; ^4^ Department of Pathology, the First Affiliated Hospital of Xinxiang Medical University, Xinxiang, Henan, China; ^5^ Department of Pathology, the Third Affiliated Hospital of Xinxiang Medical University, Xinxiang, Henan, China

**Keywords:** isoliquiritigenin, gut, microbiota, AOM/DSS, colitis-associated colorectal cancer

## Abstract

Imbalances in intestinal bacteria correlate with colitis-associated colorectal cancer (CAC). Traditional Chinese medicines have been used to adjust the gut microbiota, and isoliquiritigenin (ISL), a flavonoid extracted from licorice, has shown antitumor efficacy. In this study, the effects of ISL on CAC development and the gut microbiota were evaluated using an azoxymethane and dextran sulphate sodium (AOM/DSS)-induced mouse model of CAC (CACM). Histopathological analysis suggested that ISL reduced tumor incidence *in vivo*. Moreover, high-throughput sequencing and terminal restriction fragment length polymorphism (T-RFLP) studies of the bacterial 16S rRNA gene revealed that the structure of the gut microbial community shifted significantly following AOM/DSS treatment, and that effect was alleviated by treatment with high-dose ISL (150 mg/kg). Compared to the microbiota in the control mice (CK), the levels of *Bacteroidetes* decreased and the levels of *Firmicutes* increased during CAC development. ISL reversed the imbalance at the phylum level and altered the familial constituents of the gut microbiota. Specifically, the abundance of *Helicobacteraceae* increased after treatment with high-dose ISL, while the abundance of *Lachnospiraceae* and *Rikenellaceae* decreased. At the genus level, ISL reduced the abundance of opportunistic pathogens (*Escherichia* and *Enterococcus*), and increased the levels of probiotics, particularly butyrate-producing bacteria (*Butyricicoccus*, *Clostridium,* and *Ruminococcus*). Thus, ISL protects mice from AOM/DSS-induced CAC, and ISL and the gut microbiota may have synergistic anti-cancer effects.

## INTRODUCTION

Colorectal cancer (CRC) is a relatively common cancer that has a high mortality [1]. Prolonged periods of chronic colitis significantly increase the risk of CRC and early metastasis [2–3]. CRC occurs in the intestinal tract, which is often described as the “neglected endocrine organ” where more than 10^14^ microbes live. Interestingly, the number of microbes in gut is ten times higher than the number of human cells [4–5]. Dysbiosis of the gut microbiota has been associated with gastrointestinal diseases such as inflammatory bowel disease (IBD), type 2 diabetes, obesity, CRC, and other metabolic diseases [6–7].

Using quantitative PCR, Sobhani *et al.* demonstrated that the ratio of *Bacteroides* to *Prevotella* was significantly increased in CRC patients [8]. The abundance of probiotics such as *Bifidobacterium*, *Lactobacillus*, and *Ruminococcus* were reduced in CRC patients [9]. Arthur *et al.* found that *polyketide synthase* genotoxic islands in the commensal *Escherichia coli NC101* had a larger carcinogenic effect in the presence of intestinal inflammation [10]. Bacteria not only induce carcinogenesis, but can also produce metabolites that influence CRC progression [11]. Some metabolites including acetate, propionate, and butyrate may inhibit CRC while deoxycholic acid and lithocholic acid were shown to directly promote carcinogenesis [12]. Short chain fatty acids, particularly butyrate, can suppress inflammation and prevent CRC through several signaling pathways [13].

Many animal CRC models have been established to investigate the association between gut microbes and CRC initiation and progression. The models are based on both genetic engineering (e.g. *Apc^Min/+^*, *Muc2*^−/−^, and IL-10^−/−^ mice [14–16]) and chemical stimulation (e.g. 1, 2-dimethylhydrazine [1, 2-DMH]-treated mice and azoxymethane and dextran sulphate sodium (AOM/DSS)-induced mice [17–18]). The AOM/DSS-induced mouse model of CAC (CACM) is the most accepted CAC animal model [19–20].

Isoliquiritigenin (ISL) is a flavonoid extracted from liquorice that has anti-inflammatory and antioxidant properties [21–22]. At specific doses, ISL could reduce mouse morbidity during influenza virus infection by suppressing the inflammatory response and inhibiting viral replication [23]. ISL could also act as an anti-cancer agent by inhibiting DNA topoisomerase during glioma cell growth [24]. Previous studies have demonstrated that ISL blocks M2 macrophage polarization in the colitis-associated tumorigenesis by down regulating PGE2 and IL-6 [25]. However, the effect of ISL on gut microbiota dynamics during CAC development has not been investigated.

In this study, we used the CACM to evaluate whether ISL treatment could protect against CAC development. The anti-cancer effects of ISL were evaluated by histopathological analysis and quantification of the abundance of inflammation-associated factors/cytokines. Dynamic changes in gut bacteria were elucidated using quantitative PCR (qPCR), terminal restriction fragment length polymorphism (T-RFLP) analysis, and high-throughput sequencing of the 16S rRNA gene.

## RESULTS

### ISL prevents CAC development in BALB/c mice

**Figure 1:**
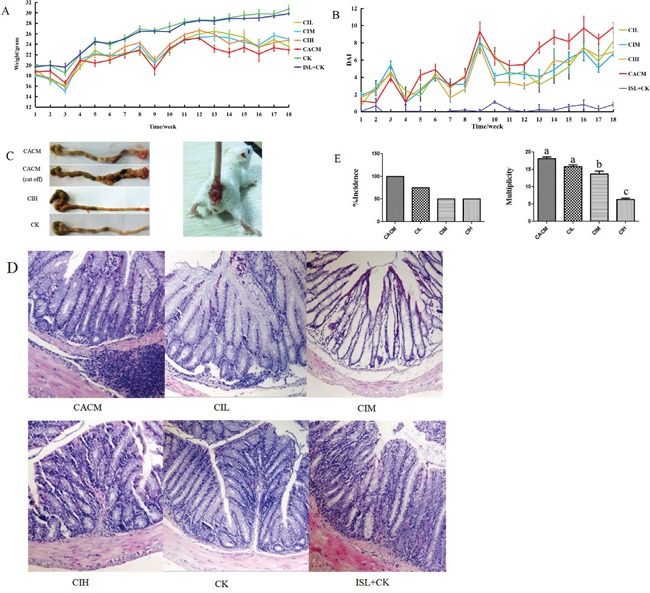
ISL protects mouse gastrointestinal tracts from AOM/DSS-induced CAC **A.** Changes in body weight. **B.** DAI based on weight loss, hematochezia, and diarrhea. **C.** Left, Macroscopic view of colon tumors at the 12^th^ week. Right, Rectal prolapse at the 12^th^ week in the CACM. **D.** Representative images of hematoxylin and eosin (HE) staining (original magnification, 100×) of mouse colon tissue at the 12^th^ week. **E.** Left, tumor incidence (percentage of tumor-bearing mice); Right, Tumor multiplicity (number of tumors per mouse). The results are presented as the mean ± standard error of the mean (SEM); n = 4 for each treatment.

The inflammation-based murine model of tumorigenesis in SPF BALB/c mice can be replicated using intraperitoneal injection of azoxymethane (AOM) and water-administered 2% dextran sodium sulfate (DSS) (Figure 1). The body weights of healthy control mice treated with 150 mg/kg ISL (ISL + CK mice) were similar to those of healthy control mice (CK mice) (Figure 1A). AOM/DSS induced CAC treatment (CACM) caused a significant loss of body weight, which was rescued by ISL treatment (AOM/DSS + 150 mg/kg ISL, CIH; AOM/DSS + 75 mg/kg ISL, CIM; AOM/DSS + 30 mg/kg ISL, CIL). We developed a disease activity index (DAI) curve to evaluate disease progression, which was based on weight, hematochezia, and stool malformation [26]. There were three peaks corresponding to the three cycles of DSS administration (in drinking water) when hematochezia and stool malformation were observed (Figure 1B). All mice in the CACM treatment group developed rectal prolapse (Figure 1C). We next evaluated the histopathological characteristics of tumor tissue samples from each group of mice (Figure 1D–1E). The multiplicity (number of tumors per mouse) in the CACM, CIL, CIM, and CIH treatment groups was 18 ± 0.35, 16 ± 0.47, 12.5 ± 0.35, and 7.5 ± 0.7, respectively. ISL decreased the incidence of cancer by 25%, 50%, and 50% at doses of 30 mg/kg, 75 mg/kg, and 150 mg/kg, respectively. Similar results were obtained at the 18^th^ week (Supplementary Figure S1).

**Figure 2:**
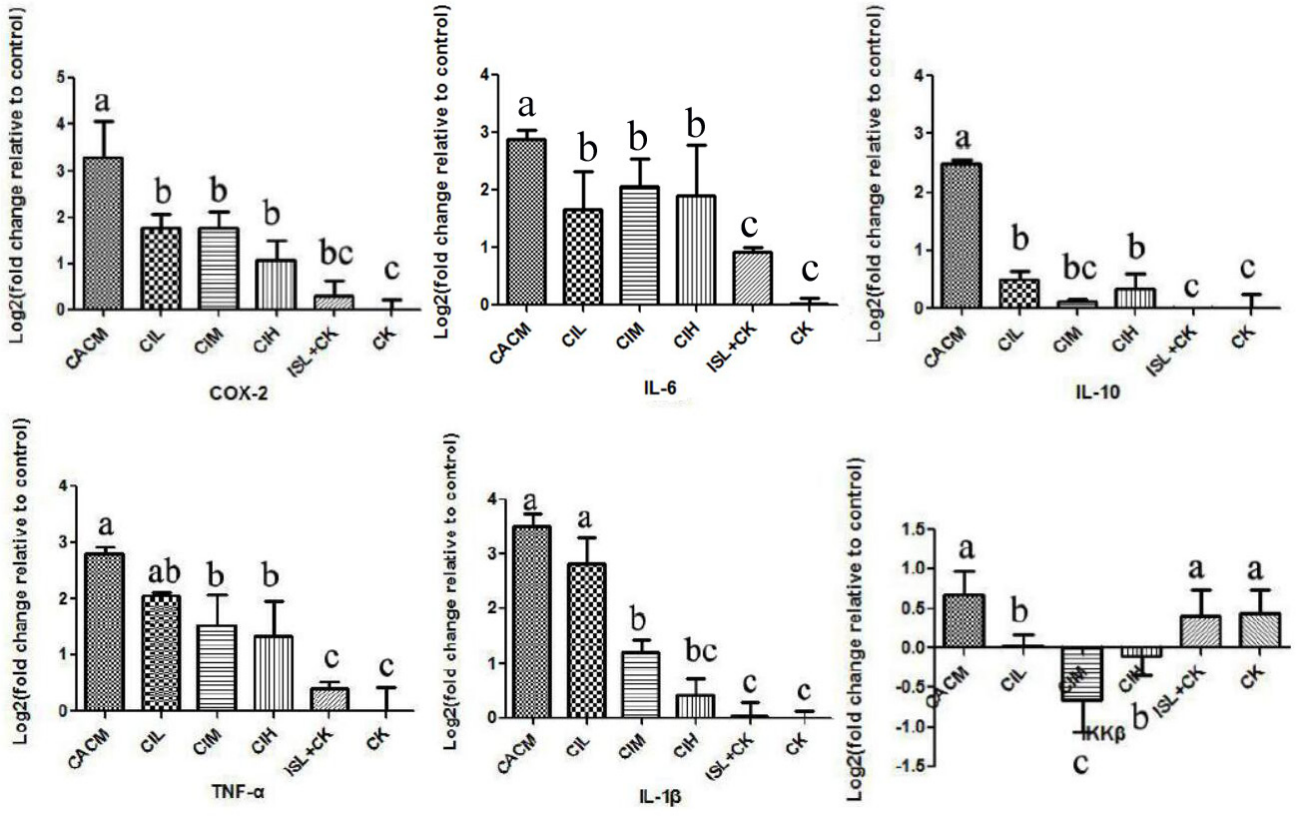
ISL inhibition of cytokine upregulation in CACM at the 12^th^ week Significant differences (*P* < 0.05) between treatments are indicated by the letters a, b, or c. The results are presented as the mean ± SEM; n = 4 for each treatment.

By the 12^th^ week, colorectal tumors formed in AOM/DSS-treated mice (CACM). To evaluate the association between the anti-cancer effects of ISL and pro-inflammatory factors/cytokines in AOM/DSS-treated mice, we quantified the levels of these factors/cytokines in mouse colon epithelial tissue samples. In the CACM, the levels of many cytokines including IL-6, IL-10, TNF-α, IL-1β, and the inflammatory factor COX-2 were increased. However, following ISL treatment, the levels decreased (Figure 2). Similar results were observed at the 18^th^ week (Supplementary Figure S2).

### Dynamic changes in the microbiota based on T-RFLP analysis

Using correspondence analysis (CA), we determined that the gut bacterial community structure changed significantly during CAC development (Supplementary Figure S3). During the 1^st^ week, the community structures were similar among all the mice (Supplementary Figure S3A). However, hematochezia and diarrhea were observed in samples from AOM/DSS-treated mice (CACM, CIL, CIM, and CIH mice) starting at the 3^rd^ week. The CACM samples were outliers compared to the other samples, which suggested that the gut bacterial community structure in the CACM differed from that in all other mice (Supplementary Figure S3B-S3D). Samples from CIL, CIM, and CIH mice were located between those of the CACM (no ISL treatment) and CK mice, which suggested that ISL protected the gut bacterial community structure from disease-associated changes. At the 12^th^ and 18^th^ week, the samples collected from CIH, ISL + CK, and CK mice were located on the same side of the 2-D biaxial and were outliers compared to all other treatment groups (Supplementary Figure S3E-S3F). These results suggested that the community structure was stable after the completion of the CACM at the 12^th^ week. The CK and CK + ISL samples clustered together throughout the experiment (Supplementary Figure S3), which indicated that ISL alone did not cause a significant shift in the gut microbiota.

### Analysis of the abundance of bacteriodes spp. and total bacteria by real-time qPCR

We next examined the abundance of *Bacteriodes spp.* and total bacteria in mouse fecal samples. Universal primers (341F/518R) for the bacterial 16S rRNA gene and *Bacteriodes spp.*-specific primers (Bfr-F/Bfr-R) were used to amplify fecal microbial DNA (Supplementary Table S1). We found that ISL significantly increased the abundance of *Bacteriodes spp.* (1.36% in CACM *vs.* 2.8% in CIH mice) at the 12^th^ week, *P* = 0.02) (Supplementary Table S2). Interestingly, compared to the CACM (no ISL treatment), a higher abundance of total bacteria was observed after low- and medium-dose ISL treatment (CIL and CIM mice). No significant difference was detected after treatment with highdose ISL (CIH mice).

### Analysis of the diversity and richness of the microbiome using 16S rRNA sequencing

To characterize the microbiome associated with CRC, high-throughput sequencing of the bacterial 16S rRNA gene was performed in fecal samples from mice in the CK, CACM (no ISL treatment), and CIH treatment groups at the 3^rd^, 6^th^, and 12^th^ weeks. Community diversity was estimated using the PD_whole_tree, Chao1, and Shannon index, and richness was evaluated based on the number of operational taxonomic units (OTUs). The Shannon index and richness were higher in the CACM than in the CK mice (Table 1). However, the Shannon index was lower in CIH mice. No significant differences were observed between the CIH and CK or between the CIH and CACM treatment groups (Table 1). ISL increased the richness of the gut microbiota in the CIH compared to the CACM and CK treatment groups. No significant differences in the PD_whole_tree or Chao1 metrics were observed between treatment groups.

**Table 1:** Bacterial diversity analyzed by high-throughput sequencing

Treatment	Richness	PD_whole_tree	Chao1	Shannon-Index
CACM	43343 ^b^	34.15 ^a^	958.53 ^a^	6.37 ^a^
CIH	51677 ^a^	37.45 ^a^	1034.16 ^a^	6.13 ^ab^
CK	41259 ^c^	31.08 ^a^	966.05 ^a^	5.77 ^b^

Note: Significant differences (*P* < 0.05) between groups are marked with the letters a, b, or c. The results are presented as the mean ± SEM; n = 4 for each treatment.

### Comparison of the gut bacterial community composition during CAC development

**Figure 3:**
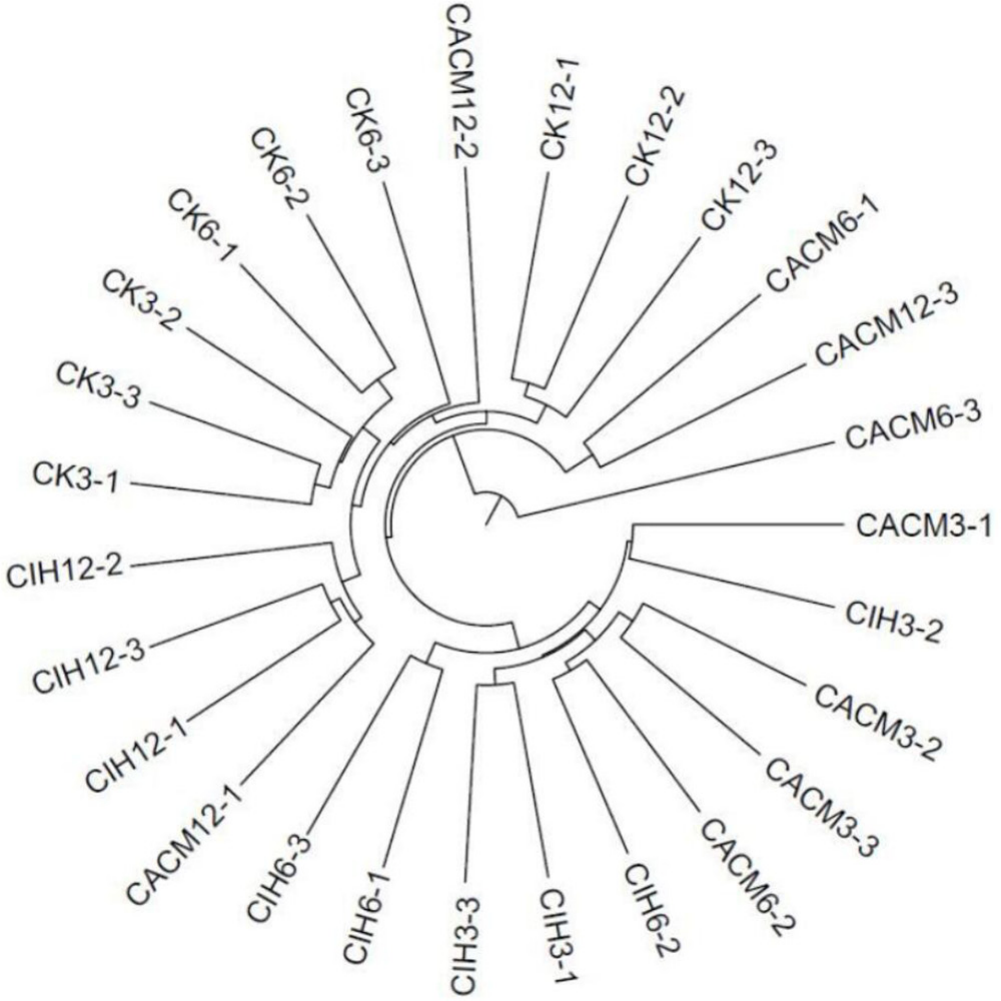
Phylogenetic tree The numbers correspond to the specimens and treatment week (e.g. CK3-1 denotes the first sample for the CK mice at the 3^rd^ week.

A phylogenetic tree was generated to examine changes in the gut bacterial community composition in response to each treatment. The greatest variations in the gut microbiota were observed at the 12 th week in the CACM (Figure 3). Notably, minimal inter-mouse variation was observed at all time points in the CK and CIH mice. The reproducibility of the samples in the CACM group was influenced by severe diarrhea and bleeding. The gut bacterial community composition changed with age in the CACM and CIH treatment groups, but remained relatively stable in the CK group. Compared to the microbiota at the 6^th^ week, the bacterial community structure markedly shifted at the 12^th^ week in the CACM and CIH mice.

**Figure 4:**
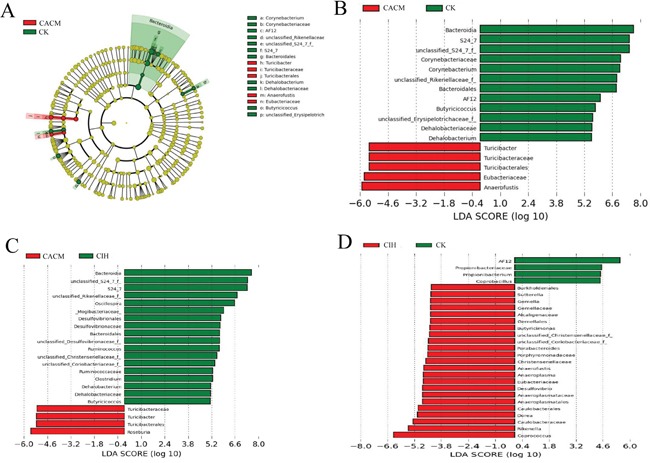
Differences in the community structures of gut microbes among the CACM, CIH, and CK mice **A.** Taxonomic representation of statistically and biologically consistent differences between CACM and CK mice. Significant differences are represented by different colors (red and green represent the core microbes in the CACM and CK treatment groups, respectively; and yellow represents microbes that were shared between the CACM and CK treatment groups. **B-D.** Histogram of the LDA scores for differentially abundant genera between the two treatment groups.

Linear discriminant analysis (LDA) coupled with effect size measurements was performed to detect core microbes in the mouse gastrointestinal tracts. The core microbes in the CK, CACM, and CIH treatment groups differed at the 12^th^ week (Figure 4). *Turicibacter*, *Turibacteraceae*, *Turicibacterales*, *Eubacteriaceae*, and *Anaerofustis* were the core microbes observed in the CACM, while *Bacteroidia*, *S24-7*, *Bacteroidales, unclassified_S24_7_f*, *Corynebacterium*, *unclassified_Rilkenellaceae_f*, *AF12*, *Butyricicoccus, unclassified_Erysipelotrichaceae_f*, *Dehalobacteriaceae*, and *Dehalobacterium* were the core microbes in CK treatment group (Figure 4A–4B). *Turicibacter*, *Turibacteraceae*, and *Turicibacterales* were also the core microbes in the CACM treatment group, whereas *Butyricicoccus*,*Dehalobacteriaceae, Dehalobacterium*, *Clostridium*, *Ruminococcus*, and *Bacteroidates* were the core microbes in the CIH treatment group (Figure 4C). ISL significantly increased microbial richness (Table 1) relative to the CACM treatment group, and resulted in a significant shift in the core microbes (Figure 4D).

**Figure 5:**
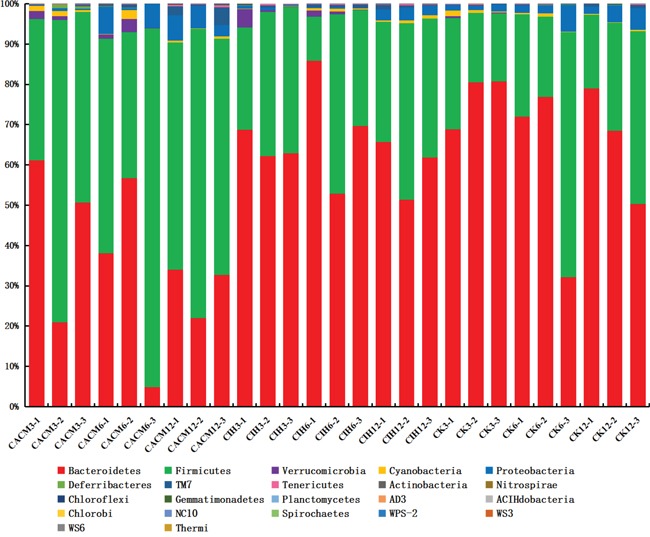
Relative abundance of the main phyla in the intestinal microbiota

To confirm the association between gut microbes and the effects of ISL treatment, we analyzed the abundance of various bacteria in fecal samples from each treatment group. At the phyla level, *Bacteroidetes* and *Firmicutes* were dominant in all of the mice (Figure 5). The levels of *Bacteroidetes* and *Firmicutes* changed dramatically in response to AOM and DSS. A significant decrease in the abundance of *Bacteroidetes* was detected at the 12^th^ week (65.76% in the CK group *vs.* 29.31% in the CACM group, *P* = 0.017). In contrast, a significant increase in the abundance of *Firmicutes* was oberseved (29.25% in the CK group *vs.* 61.69% in the CACM group, *P* = 0.02) ISL treatment did not affect the phyla distribution between CIH and CK mice (Figure 5). During CAC development, the ratio of *Firmicutes* and *Bacteroidetes* (F/B) in the CACM group was significantly higher than the ratio in the CK and CIH groups (Supplementary Figure S4).

**Figure 6:**
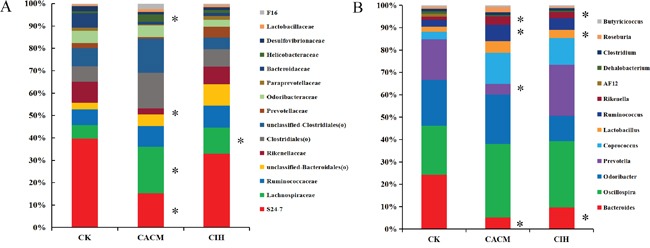
Relative abundance according to family A. and genus B. for bacteria that exceeded 1% of the total in the three treatment groups *, *P* < 0.05 compared to CK mice.

At the family level, the abundance of *S24-7* and *Rikenellaceae* decreased, while the abundance of *Helicobacteraceae* and *Lachnospiraceae* increased in the CACM compared to CK groups. ISL promoted recovery of the gut microbial community composition at the family level in the CIH group (Figure 6A). There was no significant difference between the gut microbiota in the CIH and CK groups with the exception of the *Lachnospiraceae* family (31.92% *vs.* 38.55%, *P* = 0.037).

At the genus level, 15 genera exceeded 1% of the total bacteria (Figure 6B). Consistent with the qPCR results, the abundance of *Bacteroides* decreased in the CACM group (1.14% in the CACM group *vs.* 6.18% in the CK group, *P* = 0.039). Compared to the gut microbes in the CK group, the abundance of *Prevotella* decreased (3.26% in the CK group *vs.* 0.66% in the CACM group, *P* = 0.05), while the abundance of*Coprococcus* increased (0.86% in the CK group *vs.* 3.05% in the CACM group, *P* = 0.023). ISL treatment increased the abundance of *Prevotella* in the CIH group (0.66% in the CACM *vs.* 4.68% in the CIH group) at the 12^th^ week. No significant difference in the abundanceof *Prevotella* was observed between the CIH and CK groups. Low abundance genera (< 1%) such as *Akkermansia*, *Anaeroplasma* and *Butyricimonas* were only detected in the CACM and CIH groups. In contrast, *AF12* was only detected in the CK group. The abundance of three genera (*Lachnospiraceae(f)*,*unclassified-S24-7(f)* and *Escherichia*) were significantly increased in the CACM group compared to the CK and CIH groups. Significant changes among the genera at the 12^th^ week are shown in Table 2. A heatmap based on genera abundance is shown in Supplementary Figure S5.

**Table 2:** Genera that differed significantly between CACM and CK mice, or CIH and CK mice

Phylum	Genus	Relative abundance (%)		Range (%)		P-value	Direction of change
		CACM	CK	CACM	CK		
*Bacteroidetes*	*AF12*	0	0.32	0	0.21 - 0.41	0.0053	↓
*Bacteroidetes*	*Bacteriodes*	1.14	6.18	0.23 - 2.48	3.66 - 10.7	0.039	↓
*Bacteroidetes*	*unclassified-S24-7(f)*	14.5	38.55	9.57 - 23.6	26.31 - 52.65	0.05	↓
*Bacteroidetes*	*Prevotella*	0.66	3.26	0.011 - 1.85	2.10 - 3.07	0.05	↓
*Verrucomicrobia*	*Akkermansia*	9.00E-03	0	7.2e-3 - 9.91e-3	0	0.00059	↑
*Proteobacteria*	*Escherichia*	0.15	5.00E-03	0.108 - 0.18	2.5e-3 - 8.3e-3	0.0031	↑
*Bacteroidetes*	*Butyricimonas*	9.30E-02	0	1.4e-2 - 6.8e-2	0	0.02	↑
*Firmicutes*	*Lachnospiraceae (f)*	30.4	11.26	25.51 - 37.92	8.61 - 15.20	0.012	↑
*Firmicutes*	*Coprococcus*	3.05	0.86	2.42 - 4.08	0.52 - 1.49	0.023	↑
*Firmicutes*	*Anaerotruncus*	3.30E-02	0.0086	2.6e-2 - 4.1e-2	2.5e-3 - 8.3e-3	0.027	↑
		**CIH**	**CK**	**CIH**	**CK**		
*Bacteroidetes*	*AF12*	0	0.32	0	0.21-0.41	0.0053	↓
*Actinobacteria*	*Propionibacterium*	5.20E-04	2.20E-03	0 - 1.6e-3	2.1e-3 - 2.45e-3	0.033	↓
*Bacteroidetes*	*Bacteriodes*	1.4	6.18	0.39 - 2.42	3.66 - 10.7	0.042	↓
*Bacteroidetes*	*Butyricimonas*	9.20E-03	0	7.1e-3 - 0.011	0	0.0016	↑
*Proteobacteria*	*Sutterella*	3.60E-02	0.016	3.2e-2 - 4.0e-2	1.5e-2 - 1.8e-2	0.0017	↑
*Tenericutes*	*Anaeroplasma*	2.70E-02	0	0.018 - 1.6e-2	0	0.0063	↑
*Firmicutes*	*Coprococcus*	3.16	0.86	2.68 - 3.72	0.52 - 1.50	0.0064	↑
*Firmicutes*	*Anaerofustis*	1.40E-02	0.00082	9.0e-3 - 1.8e-2	0 - 2.5e-3	0.0085	↑
*Firmicutes*	*unclassified-Christensenellaceae(f)*	9.70E-03	0.0015	5.3e-3 - 1.3e-2	0 - 4.4e-3	0.036	↑
*Bacteroidetes*	*Parabacteroides*	3.70E-02	0.0074	1.9e-2 - 5.2e-2	4.4e-3 - 1.0e-2	0.039	↑
*Firmicutes*	*Gemella*	2.00E-02	0.00069	7.1e-3 -2.5e-2	0 - 2.1e-3	0.04	↑

*Coprococcus*, *Butyricimonas*, *Roseburia*, *Clostridium*, *Ruminococcus,* and *Butyricicoccus* were the butyrate-producing bacteria identified in this study. The total abundance of butyrate-producing bacteria was 16.22% in the CACM group, 13.9% in the CIH group, and 6.03% in the CK group. *Butyricimonas* was only detected in the AOM/DSS-induced CAC mice (0.07% in the CACM group and 0.0082% in the CIH group). The abundance of *Roseburia* was slightly higher in the CACM (0.68%) compared to CIH (0.13%) and CK (0.2%) groups. The abundance of *Coprococcus* was higher in the CACM (3.05%) and CIH groups (3.16%) compared to the CK group (0.86%). Finally, the abundance of *Butyricicoccus*, *Clostridium,* and *Ruminococcus* was higher in the CIH group than in the CACM and CK groups. Changes in butyrate-producing bacteria at the 12^th^ week are shown in Supplementary Table S3.

## DISCUSSION

Previous studies have indicated that bacteria are involved in the pathogenesis of colon cancer. For example, Newman *et al.* found that *Citrobacter rodentium* promoted colon cancer in *Apc^Min/+^* mice, and Apidianakis *et al.* demonstrated a synergistic relationship between intestinal bacteria and genetic predisposition to intestinal dysplasia [14, 27]. Imbalances in microbiota can promote colon tumorigenesis through many pathways. The enterotoxigenic *Bacteroides fragilis* causes colitis, colonic hyperplasia, and tumor formation through activation of Stat3- and TH17-dependent pathways [28]. Additionally, vancomycin-sensitive bacteria induced colon inflammation and DNA damage by attracting neutrophils to damaged colon tissue, which promoted tumor formation [29]. In the present study, imbalances in the microbiota were observed in the CACM. Treatment with ISL alleviated the imbalances, reduced inflammation, and inhibited CAC development.

Following ISL treatment, the abundance of *Helicobacteraceae* decreased while the abundance of *Lachnospiraceae* and *Rikenellaceae* increased. These changes were consistent with previous studies both in animal models and in patients. The abundance of *Helicobacteraceae* increased in IBD patients [30], while the abundance of *Rikenellaceae* decreased in *Muc2*^−/−^ mice that spontaneously developed CAC [31]. Zackular *et al.* found reduced *Lachnospiraceae* in CRC patients. Some OTUs belonging to *Lachnospiraceae* may help to maintain healthy gastrointestinal tracts and could be tools to assess gut health [32]. Therefore, the increases in *Lachnospiraceae* and *Rikenellaceae* abundance could modify the gut environment and enhance the antitumor efficacy of ISL.

Previous studies have demonstrated a reduction in the abundance of *Turicibacter* in the gastrointestinal tracts of mice with colitis (DSS-induced and IL-22-deficient mice) [33–34]. However, we observed an increase in *Turicibacter* in the CACM. The increase in *Turicibacter* could have been induced by AOM treatment. In contrast, the abundance of *Turicibacter* was reduced in the CIH mice, which had a lower incidence of colon tumorigenesis. Thus, *Turicibacter* could be a tool to detect health status of the gut.

Consistent with previous studies [35–36], we confirmed that the abundance of *Bacteroidetes* decreased while the abundance of *Firmicutes* increased during CAC development. ISL treatment prevented disease-induced changes in the gut microbial community structure. The discrepancy in *Bacteroidetes* resulted in significant differences between the CACM and the CK mice. Zackular et al. found that the abundance of *Prevotella* (*Bacteroidetes)* decreased during CAC development [37]. We also observed a decrease in *Prevotella* in the CACM compared to CK group. After treatment of CACM with ISL, the abundance of *Prevotella* increased to a normal level. No significant differences were observed compared to the CK group (4.28% in the CIH group *vs.* 3.26% in the CK group, *P* = 0.74), which suggested that ISL could increase the abundance of *Prevotella* in the CACM.

Increasing evidence suggests that gut microbial metabolites are crucial for the maintenance of health [38]. Imbalances in butyrate-producing bacteria have been commonly observed in CAC patients [39–41]. Previous studies reported that butyrate had potent activity against CRC. It reduced oxidative damage to DNA, induced apoptosis in cells with DNA damage, and inhibited tumor cell growth [42]. The increase of butyrate-producing bacteria in CIH mice suggested that ISL had the ability to increase some butyrate-producing bacteria in the gut (e.g. *Butyricicoccus*, *Clostridium,* and *Ruminococcus*). *Butyricicoccus* enhanced intestinal epithelial barrier function and protected the gastrointestinal tracts of CAC patients [43–45]. *Clostridium* and *Ruminococcus* were the core microbes detected in the CIH treatment group, which suggests that they may play a key role in maintaining normal microbial balance.

*Akkermansia* was detected in fecal samples from CACM and CIH mice, but not the CK mice. Previous studies have indicated that the abundance of *Akkermansia* was positively correlated with colonic tumor multiplicity and size [37, 46], and that the abundance of *Akkermansia* in the gut was significantly increased in CRC patients [39, 47] *Akkermansia municiphila* is a component of the healthy gut microbiome and a potential probiotic, but it was positively correlated with the ratio of colon cancer [48]. We suspect it may be correlated with the abnormal gut environment induced by colitis and CRC. The abundance of *Escherichia* and *Enterococcus*, which are both opportunistic pathogens, was increased in CAC patients [49–50]. Consistent with these data, we found that the abundance of *Enterococcus* was also increased in CACM compared to CK mice (0.011% *vs.* 6.9E-4%), and was below the detection limit in CIH mice. The substantial reduction in the abundance of *Enterococcus* in CIH mice could be related to the anti-cancer effects of ISL. ISL may inhibit CAC development by reducing the abundance of some opportunistic pathogens.

ISL treatment is an effective means of controlling infection induced by certain types of bacteria and viruses. For example, Feldman *et al.* demonstrated that ISL has antibacterial activity against three major periodontopathogens: *Porphyromonas gingivalis*, *Fusobacterium nucleatum*, and *Prevotella intermedia* [51]. Moreover, the combination of ISL and oxacillin significantly lowered the systemic microbial burden of methicillin-resistant *Staphylococcus aureus* in the blood, liver, kidney, lung and spleen compared to ISL or oxacillin alone, as well as untreated controls [52]. However, the mechanisms underlying these effects are unclear. Some studies have reported that ISL suppresses inflammation through inhibition of nuclear factor-κB activation [53–57]. However, further studies are needed to fully test this hypothesis.

In conclusion, we have confirmed that ISL has anti-CAC effects. The composition of the gut microbiota in the CACM was restored upon ISL-treatment. The abundance of opportunistic pathogens were reduced (*Escherichia* and *Enterococcus*), while the abundance of *Prevotella*, *Butyricicoccus*, *Clostridium*, and *Ruminococcus* was elevated in the modified microbiota. These bacteria may cooperate with ISL to inhibit CAC development. Our study provides new evidence that traditional Chinese medicines may prevent CRC, in part through regulating the gut microbiota.

## MATERIALS AND METHODS

### Animals and reagents

Six-week-old male BALB/c mice (18–20g) were purchased from Vital River Laboratory Animal Technology Co. Ltd. (Beijing, China). All animals were housed in plastic cages (with eight mice/cage) under controlled conditions (humidity [55 ± 5%], light [12 h light/dark cycle], and temperature [23 ± 2°C]). AOM was purchased from Sigma-Aldrich (St. Louis, MO, USA) and DSS was purchased from MP Biomedicals (molecular weight: 36–50 kDa, MP Biomedicals, Santa Ana, CA, USA). ISL was purchased from Melone Pharmaceutical (molecular weight: 256.25, Dalian, China). Different doses of ISL (30, 75, and 150 mg/kg) were dissolved in 0.5% sodium carboxymethyl cellulose (CMCC-Na) solution. AOM was dissolved in normal saline to a final concentration of 0.5 mg/mL.

### Experimental procedures

**Figure 7:**
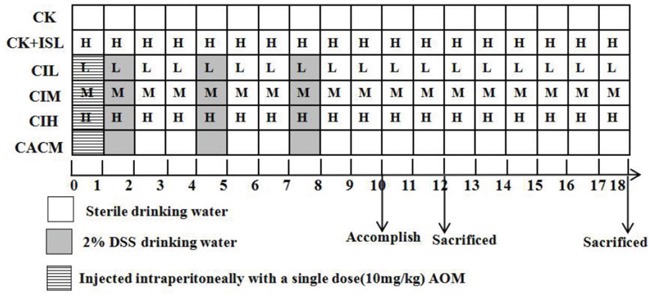
Experimental protocol Different doses of ISL are represented by the letters in the squares, (H, M, and L correspond to ISL doses of 150 mg/kg, 75 mg/kg, and 30 mg/kg, respectively). Mice in each treatment group were sacrificed at the 12^th^ and 18^th^ week based on the model.

Forty-eight six-week-old male BALB/c mice were divided into six groups: blank control treatment (CK, n = 8), ISL control treatment (ISL + CK, n = 8), AOM/DSS-induced CACM only (n = 8), and three groups of CACM mice treated with different dosages of ISL (CIL [30 mg/kg], CIM [75 mg/kg], and CIH [150 mg/kg) (n = 8 in each treatment). CK and ISL + CK mice were given sterile drinking water and fed a standard rodent chow diet for 12 or 18 weeks. The procedure for generating the CACM is shown in Figure 7. The mice were injected intraperitoneally with a single dose of AOM (10 mg/kg) on the first day. One week after AOM injection, three experimental courses of DSS were administered. For each course, the mice (CACM) were given drinking water containing 2% DSS for one week followed by sterile drinking water for two weeks. For ISL treatment, different doses of ISL (30, 75, and 150 mg/kg) were administered intragastrically six times per week starting on the first day of the study. ISL + CK mice were gavaged with 150 mg/kg ISL six times per week without AOM/DSS treatment. Animal weights were evaluated and recorded at the end of each week.

### Feces collection and bacterial DNA extraction

Fecal samples were collected at the end of each week and stored at -80°C. Bacterial DNA was extracted with the QIAamp DNA Stool Mini Kit (Qiagen, Hilden, Germany) according to the manufacturer's instructions. The quality and quantity of the DNA was evaluated by 1% (w/v) agarose gel electrophoresis in 0.5 mg/mL ethidium bromide and Nano Drop 2000 ultraviolet spectrophotometry.

### T-RFLP analysis

To evaluate the gut microbiota, feces were collected from all mice at the 1^st^, 3^rd^, 6^th^, 12^th^, and 18^th^ week and T-RFLP analysis performed. Bacterial DNA was amplified with bacterial 16S rRNA gene-specific primers: 8F (5’-FAM-AGAGTTTGATCATGGCTCAG-3’) and 1492R (5’-GGTTACCTTGTTACGACTT-3’) [58], which included a FAM label at the 5’end of the 8F primer. The PCR products were purified with an agarose gel recovery kit (DP214, Tiangen, China) according to the manufacturer's instructions. Restriction digests were performed with *MspI* (*Hpa*, *Hap II*) (Takara, Dalian, China) according to the manufacturer's instructions. PCR products (20 μL) were incubated for 4 hr at 37°C followed by 80°C for 20 min. The fragments (T-RFs) were desalinated by ethanol precipitation and then mixed with an internal size standard (LIZ500) at 95°C for 5 min. The fragments were sequenced with a DNA Sequencer in the range of 50–1000 bp (ABI PRISM 3700, USA) and the results analyzed using the Peak Scanner (v1.0) and Gene Marker 2.20 software.

### High-throughput sequencing of 16S rRNA

Based on the results of the T-RFLP analysis, 16S rRNA high-throughput sequencing was performed on fecal samples from CK, CACM, and CIH mice collected at the 3^rd^, 6^th^, and 12^th^ weeks. Genomic DNA was extracted from fecal samples using the QIAamp DNA Stool Mini Kit (Qiagen) according to the manufacturer's instructions and evaluated by 1% agarose gel electrophoresis. Genomic DNA was then amplified in 50 μL triplicate reactions with bacterial 16S rRNA gene (V3-V5 region)-specific primers: 338F (5’-ACTCCTACGGGAGGCAGC-3’) and 806R (5’-GG ACTACHVGGGTWTCTAAT-3’) [59]. The reverse primer contained a sample barcode, and both primers were connected with an Illumina sequencing adapter. PCR products were purified and the concentrations adjusted for sequencing on an Illumina Miseq PE300 system (OEbiotech Co., Ltd., Shanghai, China).

### Histopathological analysis

After the mice were sacrificed, colon specimens were dissociated and washed with cold PBS, cut off approximately 3 mm piece and fixed in 10% formaldehyde. The remaining colon tissue was used to isolate enterocytes. The fixed tissues were embedded in paraffin, sectioned, and stained for histopathological analysis [58].

### Reverse transcription qPCR

Mouse colonic epithelial cells were collected using published protocols [60–63]. Total mRNA was extracted using the Trizol reagent and colonic epithelial cytokines evaluated by reverse transcription qPCR (RT-qPCR). The results were analyzed using the ΔΔCt method [64]. Fecal DNA was amplified with bacterial 16S rRNA-specific primers and the relative quantities of total bacteria and *Bacteroides spp.* analyzed. Target gene copy number was determined by comparison to a standard curve. PCR reactions were performed using the StepOne System (ABI). The primer sequences and qPCR amplification protocol are shown in Supplementary Table S4.

### Bioinformatics and statistical analysis

For T-RFLP analysis, the abundance of T-RFs (< 1%) with lengths < 30 bp were filtered. T-RFs that differed by ± 1 bp were combined into a single T-RF. CA of the bacterial community structures was performed using the Canoco for Windows 4.5 software.

For high-throughput sequencing, raw reads were processed with the Trimmomatic software. First, pair reads were merged according to their overlap. The sequences were then filtered according to the barcode and primer sequences (the barcodes could not be mispaired and the highest number of mispaired primer sequences was two. The optimized sequences were clustered into OTUs with 97% similarity using Usearch (version 7.1 http://drive5.com/uparse/). The OTUs were used to estimated community diversity and richness. The alpha diversity analysis was performed with mothur (version v. 1.30.1 http://mothur.org/). The Shannon index, PD_whole_tree, and Chao1 were used to estimate community diversity, while richness was calculated based on the number of OTUs. A heatmap based on Bray-Curtis was made with the R vegan kit (R package 2.7.1).

### Statistical analyses

Microbial taxonomy features were analyzed using Mann-Whitney tests (SPSS 19.0, Chicago, IL, USA). Significant differences in the diversity index, richness, cytokine abundance, and tumor multiplicity were identified using repeated measures ANOVA with Tukey's honestly significant difference (HSD) post hoc test in SPSS 19.0. Statistical tests were two-sided and a *P* < 0.05 was considered significant.

## SUPPLEMENTARY MATERIALS FIGURES AND TABLES


