# Supplementary material for: Isoliquiritigenin decreases the incidence of colitis-associated colorectal cancer by modulating the intestinal microbiota
Source: Oncotarget. 2016 Nov 15;7(51):85318–31. doi: 10.18632/oncotarget.13347 (PMC5356739; doi:10.18632/oncotarget.13347)
Supplement: Supplementary file 1 [file oncotarget-07-85318-s001.pdf]

Isoliquiritigenin decreases the incidence of colitis-associated colorectal cancer by modulating the intestinal microbiota

SUPPLEMENTARY FIGURES AND TABLES

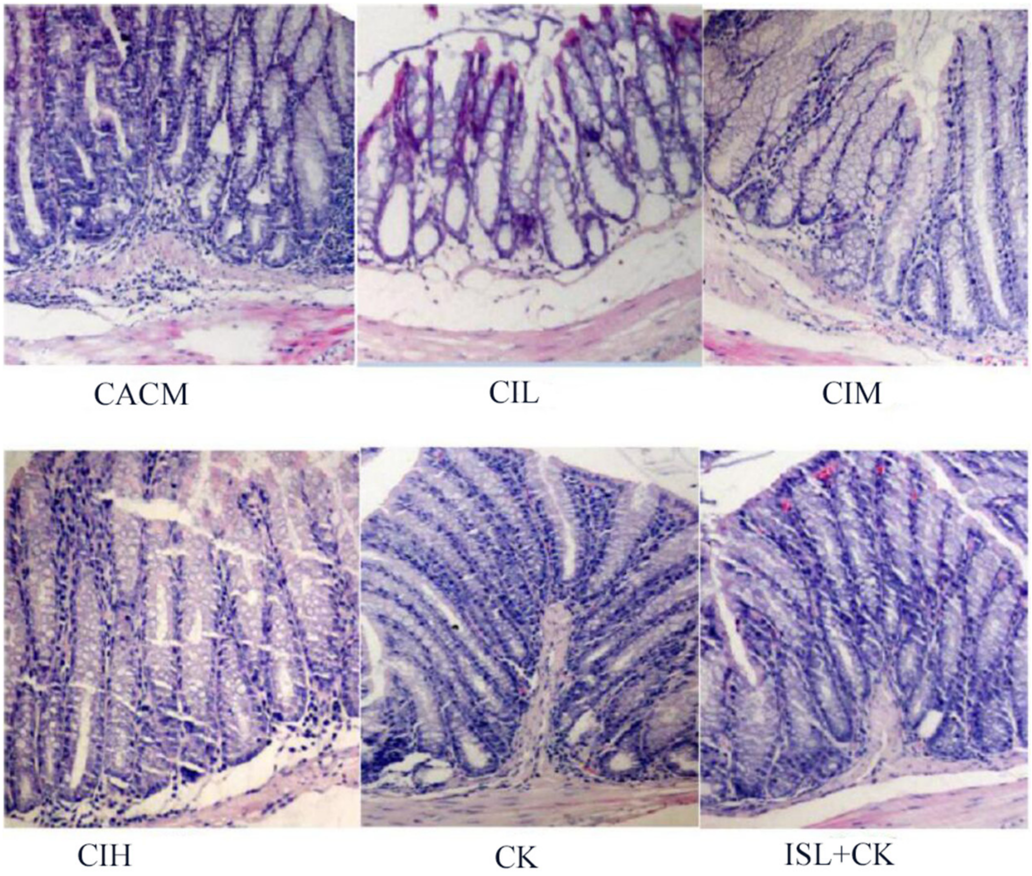

Supplementary Figure S1: Representative images of HE stained (original magnification, 100×) mouse colon tissue samples at the 18th week.

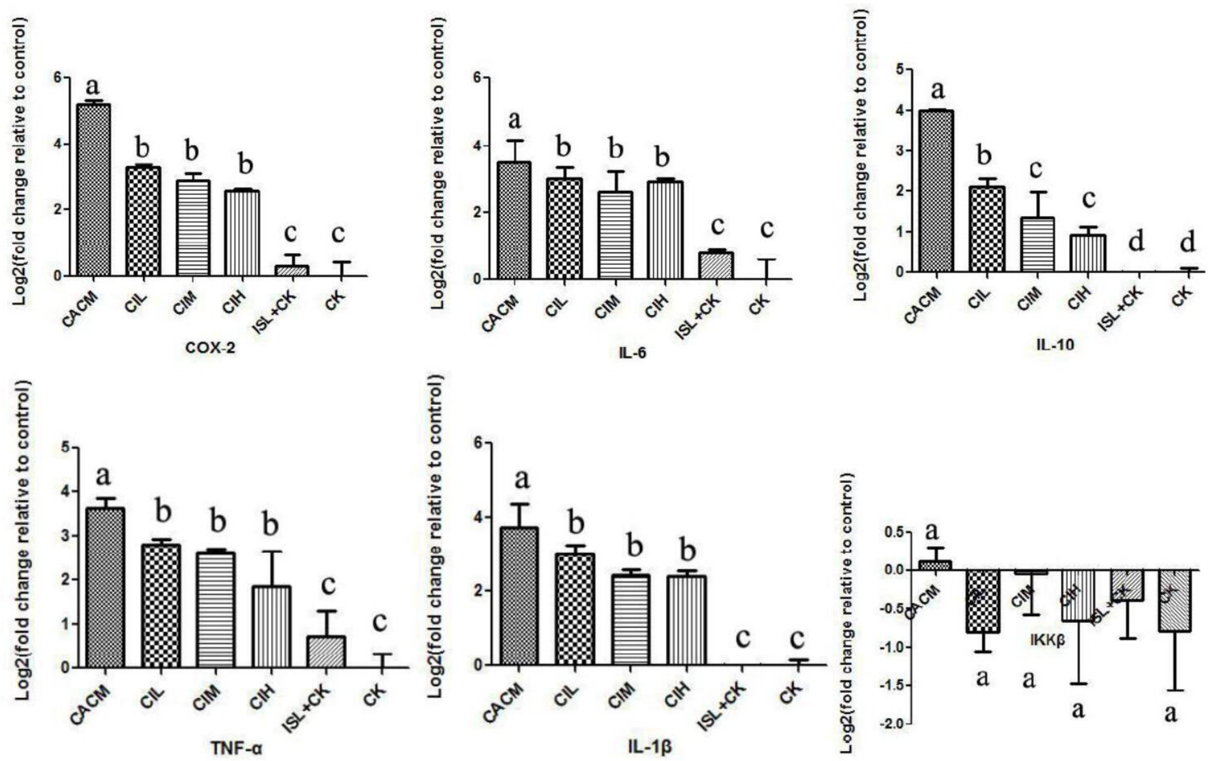

Supplementary Figure S2: ISL inhibited cytokine upregulation in CACM at the 18th week. Significant differences ($P < 0.05$) between the treatments are indicated by the letters a, b, or c. The results are presented as the mean ± SEM; n = 4 for each treatment.

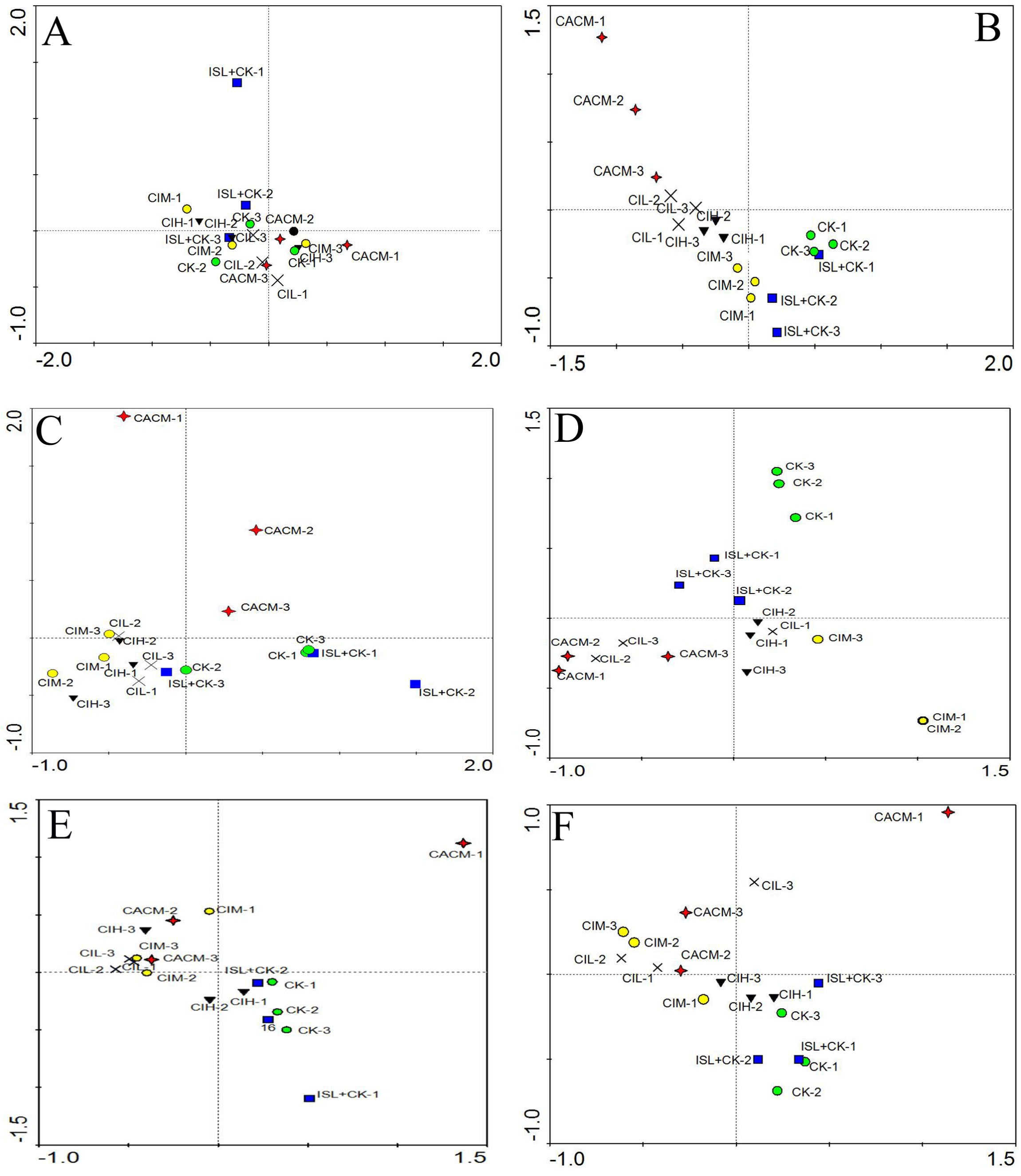

Supplementary Figure S3: CA analysis of the microbial community. The red stars represent samples in the CACM treatment group; crosses, CIL; yellow circles, CIM; black triangles, CIH; green circles, CK; blue squares, CK + ISL. Each treatment group has three samples. **A-F.** CA analysis of the microbiota at the 1st, 3rd, 6th, 9th, 12th, and 18th weeks.

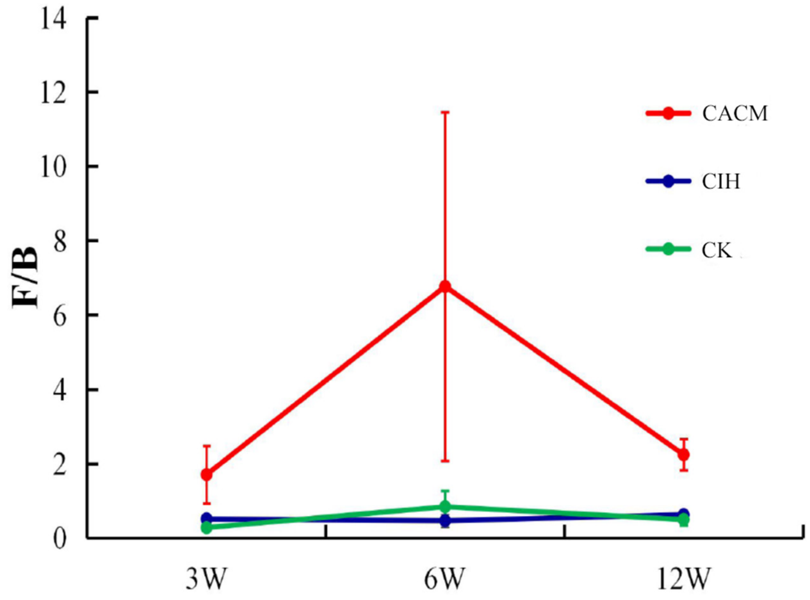

Supplementary Figure S4: Dynamic shift in the ratio of *Firmicutes* to *Bacteroidetes* (F/B). The results are presented as the mean ± SEM; n = 4 for each treatment.

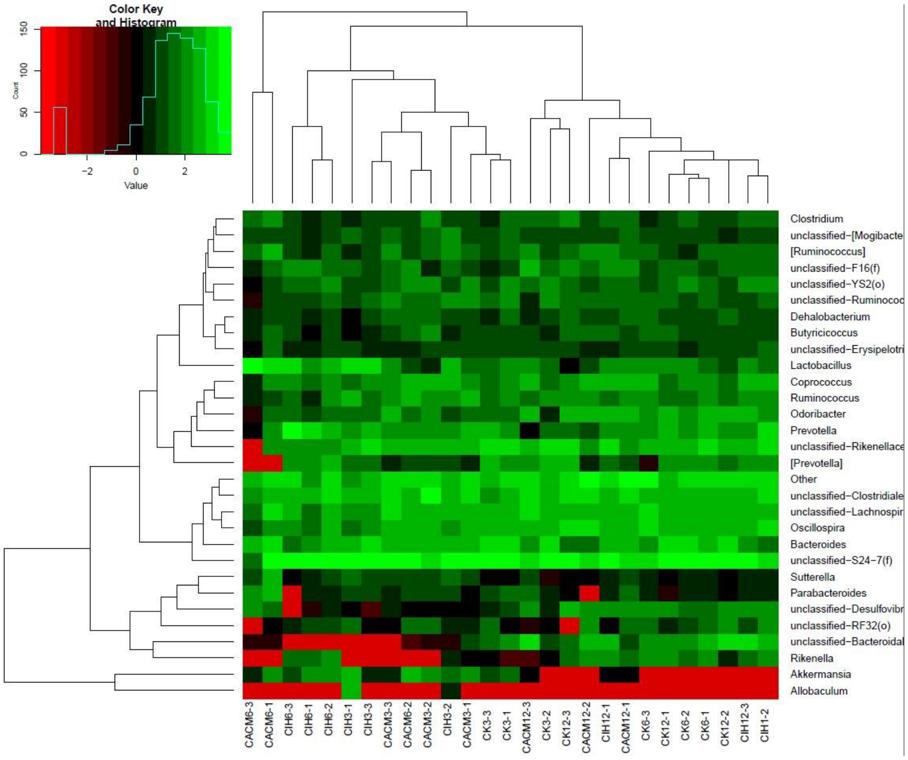

Supplementary Figure S5: Heatmap. The numbers correspond to the sample information (e.g. CK3-1 denotes the first sample from CK mice at the 3rd week.

Supplementary Table S1: The bacterial primer sequences and qPCR amplification procedures

Target bacterial group	Primers	Primer sequences	References	Amplification conditions
Total bacteria	341F	5′- CCT ACG GGA GGC AGC AG-3′	(Nadkarni et al., 2002)	Initial denaturation at 95°C for 4 min; 40 cycles of denaturation at 95°C for 15 s, and annealing/extension at 60°C for 1 min.
	518R	5′-ATT ACC GCG GCT GCT GG-3′		
Bacteroides spp.	Bfr-F	5’-CTG AAC CAG CCA AGT AGC G-3’	(Louis & Flint, 2007)	Initial denaturation at 95°C for 2 min; 40 cycles of denaturation at 95°C for 30 s, annealing at 52°C for 30 s, and extension at 72°C for 1 min.
	Bfr-R	5’-CCG CAA ACT TTC ACA ACT GAC TTA-3’		

Supplementary Table S2: Analysis of bacterial abundance in mouse fecal samples by qPCR

	Bacteriodes spp. Log(copies/ng)	Total bacterial Log(copies/ng)	*Bacteriodes spp.* relative to total bacteria (%)
CIL	5.05 ± 0.01 [a]	6.56 ± 0.08 [a]	3.17 ± 0.47 [b]
CIM	4.93 ± 0.07 [b]	6.61 ± 0.05 [a]	2.09 ± 0.15 [c]
CIH	4.76 ± 0.15 [bc]	6.35 ± 0.05 [b]	2.80 ± 0.76 [bc]
CACM	4.49 ± 0.10 [d]	6.36 ± 0.10 [b]	1.36 ± 0.04 [d]
CK	4.84 ± 0.02 [c]	6.25 ± 0.07 [b]	4.25 ± 0.82 [a]
CK+ISL	5.03 ± 0.02 [a]	6.55 ± 0.02 [a]	3.50 ± 0.24 [ab]

Note: Significant differences ($P < 0.05$) between the treatments are indicated using the letters a, b, or c. The results are presented as the mean ± SEM; n = 4 for each treatment.

Supplementary Table S3: Relative abundance of butyrate-producing bacteria

Genus	Relative abundance (%)		
	CACM	CIH	CK
Roseburia	0.68 ± 0.04	0.13 ± 0.051	0.21 ± 0.020
Butyricimonas	0.07±0.033	0.0092±0.00099	0
Coprococcus	3.10±0.088	3.20±0.57	0.86±0.17
Butyricicoccus	0.15±0.021	0.21±0.025	0.18±0.026
Clostridium	0.27±0.023	0.32±0.031	0.28±0.095
Ruminococcus	0.65±0.017	0.81±0.016	0.48±0.014
Total	9.20	13.90	6.03

Supplementary Table S4: Cytokine primer sequences

Gene name	Forward primers	Reverse primers
COX-2	TAGTCCTTCCTACCCCAATTTCC	GCACGTAGTCTTCGATCACTATC
IL-6	TAGTCCTTCCTACCCCAATTTCC	TTGGTCCTTAGCCACTCCTTC
IL-10	TGAGCAACTATTCCAAACCAGC	CGCAGCTCTAGGAGCATGTG
TNF-α	CCCTCACACTCAGATCATCTTCT	GCTACGACGTGGGCTACAG
IL-1β	GCAACTGTTCCTGAACTCAACT	ATCTTTTGGGGTCCGTCAACT
IKKβ	CTGAAGATCGCCTGTAGCAAA	TCCATCTGTAACCAGCTCCAG
GAPDH	TCTGGAAAGCTGTGGCGTGAT	GCCAGTGAGCTTCCCGTTCAG

Note: The qPCR amplification procedures were: 95°C 5s; 40 cycles of denaturing at 95°C for 15 s, and annealing/extension at 60°C for 1 min. After all cycles, dissociation curves were performed as 95°C for 15s, 60°C for 1 min, and 95°C for 15°C.
